# Jump assessment on a force plate—an approach to quantify subtle lower limb neuromuscular deficits in people with multiple sclerosis

**DOI:** 10.3389/fneur.2026.1813457

**Published:** 2026-05-08

**Authors:** Anne Geßner, Maximilian Hartmann, Heidi Stölzer-Hutsch, Katrin Trentzsch, Tjalf Ziemssen

**Affiliations:** Center of Clinical Neuroscience, Neurological Clinic, Faculty of Medicine and University Hospital Carl Gustav Carus and Centre for Tactile Internet with Human in the Loop (CeTI), TUD Dresden University of Technology, Dresden, Germany

**Keywords:** jump assessment, multiple sclerosis, neuromuscular deficits, precision rehabilitation, sensor technology

## Abstract

Neuromuscular impairments are common in people with multiple sclerosis (pwMS), often beginning subclinically in early disease stages and contributing to long-term disability. Traditional clinical tools such as the Expanded Disability Status Scale (EDSS), however, lack sensitivity for detecting subtle neuromuscular dysfunction. In recent years, jump assessment has emerged as a digital, performance-based approach to evaluate lower-limb neuromuscular function in pwMS. Vertical jump tests on a force plate provide objective, quantifiable markers of strength, coordination, and balance, allowing detection of early motor deficits even in pwMS with minimal disability and enabling functional phenotyping to guide individualized neurorehabilitation strategies. In this narrative review, we summarize current applications and future perspectives of jump assessment in multiple sclerosis and present the structured, adaptive jump protocol which combines countermovement jumps (CMJ), single-leg CMJ (SLCMJ), and the 10-s hop test (10SHT). Overall, jump assessment represents a promising approach for detecting early neuromuscular deficits and improving the long-term care of pwMS.

## 1 Introduction

Multiple Sclerosis (MS) is a chronic inflammatory autoimmune disease of the central nervous system (CNS). The multifactorial pathophysiology of MS leads to demyelination of axons in the CNS affecting every neurological functional system (1). Consequently, the clinical presentation of MS is extremely heterogeneous including deficits in neuromuscular function (i.e., interaction between the nervous and the muscular system) and decrements in mechanical function of the lower-limb muscles (i.e., muscle strength, muscle power, and explosive muscle strength) (2, 3). These neuromuscular impairments have critical implications for all levels of the International Classification of Functioning (ICF), Disability and Health model including activity level in pwMS (4). Several studies have shown that decreased neuromuscular function is associated with impaired functional ability, activities of daily living (ADL) and quality of life (2, 3, 5). In particular, ADL functions such as stair climbing, sit-to-stand ability and walking performance can be affected by reduced neuromuscular functions (6, 7). In the early stages of MS, subtle neuromuscular deficits may also occur that are not detected during clinical examination or by the patient themselves. The efficient and effective monitoring of subtle neuromuscular impairments is crucial for optimal disease-modifying and symptomatic treatment in MS (8). Evidence is accumulating to suggest that there is a period early in the course of MS when treatment is most effective, and that effective treatment during this period appears to be critical for maintaining long-term neurological function and preventing later disability (9, 10).

Among the many methods to quantify MS-related disability of neuromuscular function the Expanded Disability Status Scale (EDSS) is a typically und widely used assessment, characterized by the impairment of other different functional neurological systems (i.e., visual, brainstem, cerebellar, sensory) and ambulation on a scale from 0 to 10 (11). Krieger et al. (12) demonstrated that traditional clinical measures are insufficient for detecting sub-threshold deficits (e.g., at EDSS 0), while high-challenge tests of balance and coordination provide to be more sensitive. Another recent study has shown that highly demanding tasks compared to traditional assessments such as the EDSS or Timed 25-Foot Walk Test (T25FWT) are necessary in MS to reveal neuromuscular deficits in pwMS with low levels of disability (13). These traditional assessments have widespread acceptance in the field, but they provide limited detection of more subtle neuromuscular deficits, especially early in disease.

In addition to the studies by Kirkland et al., there is initial evidence, that pwMS without lower limb strength, coordination and sensory impairments on the EDSS require more functionally complex assessments, such as jumping, to identify motor and neuromuscular deficits below the clinical threshold of the EDSS (14, 15). Evaluating several domains (coordination, balance, proprioception and strength) in one assessment, jumps could be used to assess time-efficiently and functionally complex neuromuscular functions, identify specific deficits and implement appropriate neurorehabilitation approaches. Understanding the biomechanics of jumping could therefore a prerequisite for evaluating neuromuscular function using jumps in pwMS.

Recent advances in personalized and precision medicine in MS highlight the growing need for sensitive, multidimensional tools capable of capturing individual functional profiles and early subclinical changes (16–18). Precision rehabilitation, an emerging concept within modern MS care, aims to tailor therapeutic interventions to the specific motor, cognitive, and behavioral phenotypes of each patient. This requires objective, high-resolution biomarkers that can detect subtle alterations in neuromuscular control before they manifest as measurable disability (18–20). As part of this shift toward more individualized disease monitoring, performance-based functional tests that integrate biomechanical, sensor-derived, and patient-centred data are becoming increasingly important.

Apart from the initial work by Kirkland et al. and our own studies by Geßner et al., little is currently known about jump assessment in MS (14, 15, 21–24). Searching PubMed for relevant literature using the search terms ‘jump assessment AND multiple sclerosis’ proved to be inefficient. We therefore broadened our search to other clinical conditions in which jump assessment has been more extensively investigated, including paediatric neuromuscular diseases, sports injuries, and age-related disorders.

In this narrative review, we discuss current approaches to jump assessment in MS and their suitability for specialized clinical practice, including the necessary infrastructure. In addition, we present the vertical jump assessment protocol as an example of implementation used in our specialized MS center in Dresden as an expert-practice proposal for structured clinical implementation. We focus on critical limitations of existing procedures and potential improvements enabled by digital tools. In this context, we also highlight current and future applications of newer technologies such as smartphone apps and wearable devices to enhance patient awareness, provide easier and cheaper access and support active disease self-management.

## 2 Mechanisms and biomechanics of vertical jumps

Three main categories of jumping are commonly described: vertical jumps, drop jumps and horizontal jumps. Vertical jumps primarily measure vertical force generation and are a well-established test of lower limb strength (25, 26). In comparison to horizontal and drop jumps, which are more complex and risky, vertical jumps require less space, can be performed safely, constitute a multi-joint movement that requires complex motor coordination and are recognised as a fundamental movement skill (14, 26, 27).

**Figure 1:**
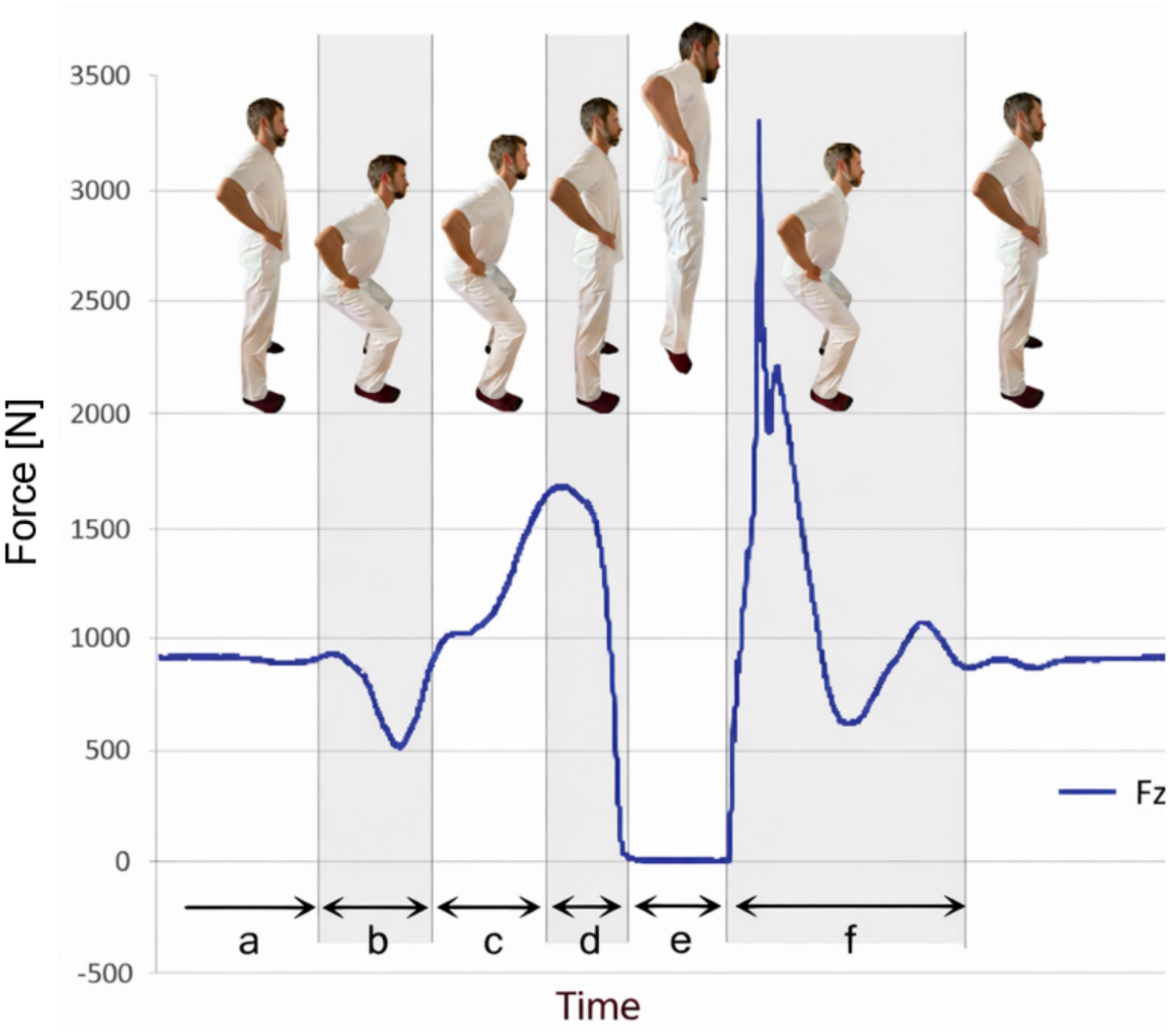
Jump phases of vertical countermovement jumps in the force-time curve. a = weight phase, b = unweighted phase, c = eccentric phase, d = concentric phase, e = flight phase, f = landing phase, Fz = resulting force.

Vertical jumps require a complex interplay of muscular, neurological and biomechanical factors. Central to these mechanisms is the activation of the stretch-shortening-cycle (SSC). The SSC is defined by a short, high-intensity eccentric contraction (muscle stretching) immediately before a rapid concentric contraction (muscle shortening) (28, 29). Muscles acting about a joint function naturally through a combination of eccentric and concentric activations (30). The muscle stretching in the eccentric phase triggers spinal reflex responses that lead to greater muscle stimulation in the concentric phase. In addition, kinetic energy can be stored in the muscle-tendon system as potential energy in the eccentric phase, which can be used in the concentric phase. As a result of this storage, concentric power can be greater in movements with SSC than in purely concentric movements (31). The SSC increases the efficiency of force production through the storage and release of elastic energy in the tendons and muscles. The neuromuscular system plays a crucial role, with motor unit recruitment and firing rates essential to maximise power output during the concentric phase (32). Studies have shown that higher levels of preactivation of muscles such as the quadriceps and calf muscles can significantly increase jump height (33). In addition, biomechanical factors such as joint angles during the jump phase, the sequence of limb movements and the optimal timing of force application contribute to the effectiveness of a vertical jump (34). Studies showed that most of the work in a vertical jump is performed by the hip (41–51%), followed by the knee (29–33%) and ankle (16–28%) (35, 36). Raffalt et al. (37) found that during the eccentric phase of Countermovement Jumps (CMJ), the knee joints performed significantly more work than the hip and ankle joints, with the hip joint also producing significantly more work than the ankle joint. During the concentric phase, more work was performed by the hip and knee joints than by the ankle joints. Research suggests that an optimal pattern of coordination between the hip, knee and ankle joints is essential for maximising jump height (38). Furthermore, jumping performance is linked with muscle morphology (39). The role of muscle fibre type composition, particularly the proportion of fast fibres, has been highlighted as a determinant of explosive power and jumping performance (30). The speed of muscle contraction is also an important factor in power generation. Aeles et al. (29) used electromyography (EMG) as well as kinematic and kinetic data to demonstrate that fast muscle contraction and increased jump power are primarily driven by the stretch–shortening cycle (SSC), particularly through the medial gastrocnemius muscle–tendon unit and the tendon’s serial elastic element. The vertical jumps with countermovements like the CMJ and SLCMJ can be divided into 6 phases (Figure 1). In the first phase (a), the patient stands still on the force plate and the body weight is measured. In the second phase (b), the patient begins a short counter-movement with hip and knee flexion in which the body weight is reduced below a threshold value of 10%. The phase ends when the body weight in the force-time curve is reached again. The third phase is the braking phase or eccentric phase (c), which is characterised by the flexion of the hips, knees and ankles until the centre of mass (COM) is at its lowest and the velocity is zero. During the braking phase, the following leg muscles work eccentrically: M. gluteus maximus, M. iliopsoas, M. quadriceps femoris and M. triceps surae. This is followed by the push-off movement or concentric phase (d). It begins with a powerful extension of the hips, knees and ankles in order to move COM upwards and push off from the force plate. The muscles that previously worked eccentrically during the braking phase now work concentrically. The time from the force plate to the highest point of the COM is referred to as the flight phase (e). The CMJ ends with the landing phase (f) when both feet touch the force plate and the starting position is reached again.

The described mechanisms and biomechanical factors are particularly relevant for the assessment of neuromuscular deficits in people with MS. Investigating specific aspects such as joint work and muscle activation patterns could help to better understand individual impairments and inform targeted therapeutic approaches.

## 3 The use of jump assessment

### 3.1 Possibilities of jump assessment in specific clinical conditions

Jump assessment has its origins in sports science and was originally developed to assess and improve the performance and technique of athletes in jumping disciplines such as high jump, basketball and volleyball (31, 40–42). In addition, vertical jump tests are widely used by strength and conditioning professionals, coaches and healthcare professionals to assess lower limb muscle strength due to their simplicity and richness of outcome information (43). By analysing the mechanics of the jump in detail, coaches and sports scientists were able to define an athlete’s profile, identify talent, monitor performance, identify predictors of performance, determine asymmetries, and indicate readiness to return from injury (25, 44, 45). These findings were crucial for optimising athletic performance and technique and to develop new training equipment and programmes that improve jumping performance.

In addition to its application in sports science, the assessment of jumping is a fundamental component of evaluating motor development, physical fitness, and overall health in children and paediatrics (46). Jumping is an important motor skill that develops in early childhood, typically beginning at the age of three and continuing to refine throughout early and middle childhood (47). This skill is critical as it reflects the development of coordination, balance and muscle strength, all of which are important for a child’s overall motor competence and physical health (46, 48). Deficits and imbalance of foot and ankle muscle strength are characteristic of many paediatric neuromuscular disorders such as Charcot–Marie–Tooth disease (CMT), Duchenne muscular dystrophy and Cerebral Palsy (49–51). To assess these neuromuscular deficits in paediatric neuromuscular disorders, jumps are used in established paediatric motor assessments such as the CMT Pediatric Scale and Gross Motor Function Classification System (52, 53). A study of Alexander et al. demonstrated that children with CMT disease exhibited impaired jump performance, characterised by a reduction in jump height, peak force and vertical impulse (54). Findings on the reductions in jumping ability in children can be useful for clinical management, including the implementation of specific and patient-centered training (54). Furthermore, to monitor age-related changes in muscle strength in the lower limbs, the assessment of jumping performance is also useful in sarcopenia, osteopenia and osteoporosis (55, 56). Jump assessments in sarcopenia can help identify muscle weakness, a key component of sarcopenia, to provide early diagnosis and monitor progression (57). Other findings included that jump power may be a better indicator of muscle performance in older people than traditional isotonic muscle strength measures. In contrast to traditional isotonic muscle strength measurement, the jump assessment measures not only muscle power but also contraction velocity (57, 58).

### 3.2 Possibilities of jump assessment in MS

Although findings from sports science and other populations help to support the biomechanical rationale of jump assessment, they cannot substitute for MS-specific validation of jump metrics and their clinical interpretation. Overall, the jump assessments provide a comprehensive assessment of lower limb neuromuscular function, independent of specific clinical conditions such as cruciate ligament injury, sarcopenia or CMT disease. Understanding how to apply these tests in various clinical contexts not only enhances the ability to assess neuromuscular function, but also deepens the understanding of their potential to identify subtle deficits in neurological conditions such as MS (14, 24). The ability to identify, specify and monitor these subtle neuromuscular impairments could be essential for optimising both disease-modifying therapies and neuro-rehabilitative interventions in MS (10, 18, 59). The jump assessment in MS may be useful not only for early detection, but also for determining the exact movement impairment during eccentric and concentric muscle activation. It is important to understand how MS affects both types of contraction, as everyday movements are a finely tuned sequence of concentric and eccentric contractions (60). Furthermore, the jump assessment measures the functional activity of the total lower limb muscle chain and provides a better simulation of everyday movements. Correlation results from the first studies investigating vertical jumps in MS suggested significant relation between EDSS and jump parameters (14, 24). These findings indicate that vertical jumps on a force plate can add comprehensive objective and metric parameters that provide important additional information about pyramidal, cerebellar and sensory function to the traditional neurostatus examination.

An overview of the currently available studies on jump assessment in pwMS is provided in Table 1.

**Table 1:** Overview of the currently available studies on jump assessment in pwMS.

Study	Jump test	Sample size	Disability range	Outcomes	Main findings	Study limitations
Kirkland et al. (15)	bipedal hopping	pwMS = 13; HC = 9; elderly = 12	2.04 ± 1.16	Jump metrics; T25FWT	pwMS (mild disability) showed intermediate performance between controls and elderly No significant differences between pwMS and other groups in most hopping measures Hop length (not walking measures) predicted disability (EDSS) (R^2^ = 0.38, *p* = 0.02)	High variability within the MS group Small sample size No comparison with direct muscle characteristics (e.g., strength, atrophy)
Geßner et al. (14)	CMJ	pwMS = 99; HC = 33	0–3.5	Temporal parameters; kinetic parameters; performance parameters	pwMS with preserved motor function show significantly reduced CMJ performance vs. HC (almost all parameters, *p* < 0.05) Jump performance declines significantly with increasing disability CMJ detects motor deficits below the threshold of clinical neurological examination	Cross-sectional study No additional isolated strength measurements to increase objectivity and validation of findings
Geßner et al. (22)	CMJ	pwMS = 164; HC = 98	1.5–2.0	Temporal parameters; kinetic parameters; performance parameters	Significant effects of age, sex, and BMI on all CMJ parameters (pwMS & HC) No significant interaction effects between group (pwMS vs. HC) and these variables Strongest effects for sex: flight time (η^2^ = 0.23), jump height (η^2^ = 0.23), positive power (η^2^ = 0.13) pwMS show lower CMJ performance vs. HC (middle-aged, normal–overweight, both sexes)	Cross-sectional study No additional confounders (demographic, clinical, imaging, biomarkers)
Geßner et al. (24)	SLCMJ	pwMS = 126; HC = 79	0–2.5	Temporal parameters; kinetic parameters; performance parameters	Significantly reduced SLCMJ performance in pwMS compared to HC Jumping performance differed significantly between the dominant and non-dominant leg, with higher effect size for pwMS. pwMS showed an even higher difference between the dominant and non-dominant leg compared to HC Significant small correlation between leg asymmetries and EDSS in pwMS.	Cross-sectional study Dominant leg defined by highest jump height Lack of additional objective measures (e.g., isokinetic dynamometry, electromyography)
Geßner et al. (23)	10SHT, CMJ, SLCMJ	pwMS = 157; HC = −	1.5–3.0	Temporal parameters; kinetic parameters; performance parameters	Overall positive patient experience with sensor-based jump assessment Best-rated aspects: staff support, time efficiency, usefulness, and therapy integration CMJ perceived as easiest and least exhausting test (*p* < 0.05)	Cross-sectional study No clinical staff evaluation included Self-developed PREM questionnaire

pwMS, people with Multiple Sclerosis, HC, healthy controls, EDSS, Expanded Disability Status Scale, T25FWT, Timed 25 Foot Walk test, 10SHT, 10 s hop test, CMJ, countermovement jump, SLCMJ, single leg countermovement jump, MS, multiple sclerosis, PREM, patient reported experience measure.

## 4 Infrastructure for jump assessment

Research on jump assessment to measure neuromuscular function needs a complex infrastructure to generate quality data. The global market offers simple to sophisticated measurement systems for jumping analysis. However, despite the exponential growth of performance monitoring technologies, force plates are still considered the gold standard for assessing valid jumping performance, as they allow sports scientists and practitioners to measure a variety of biomechanical variables with a high degree of reliability (61–63). Therefore, not only a complex infrastructure is needed but also trained medical staff to accompany and to conduct the tests as well as to analyze the data (64). A selection of potential assessment technologies for vertical jump assessment and a selection of their associated outcomes is shown below in Table 2.

**Table 2:** Selection of potential assessment technologies for vertical jump assessment.

Assessment technology	Method	Outcomes	Selection of device examples*
Video-based	a) Marker based b) Marker free	a) and b) Movement analysis, jump parameter	a) Vicon (Vicon Motion Systems Ltd), Miqus Hybrid (Qualisys AB) b) My Jump App
Force plates	Piezoelectric sensors, Strain-gauge sensors	Ground reaction force, jump parameter	AMTI (Advanced Mechanical Technology Inc.), Bertec, Kistler
Sensor floor plates	Capacitive or resistive pressure sensors	Plantar pressure distribution, temporal–spatial parameters, CoP trajectories	GAITRite Smart insoles (e.g., Moticon)
Wearable sensors	3-dimensional accelerometer sensors	movement analysis, acceleration, velocity,	Opal sensor (APDM, Inc.)
Optoelectronic systems	Light beam-based	jump height, fight time	OptoJump Next (Microgate)
Electromyography	Surface electrodes or fine-wire electrodes	Muscle activation pattern	Delsys, Noraxon

* All devices are presented as a selection of examples only and do not constitute a specific recommendation.

**Figure 2:**
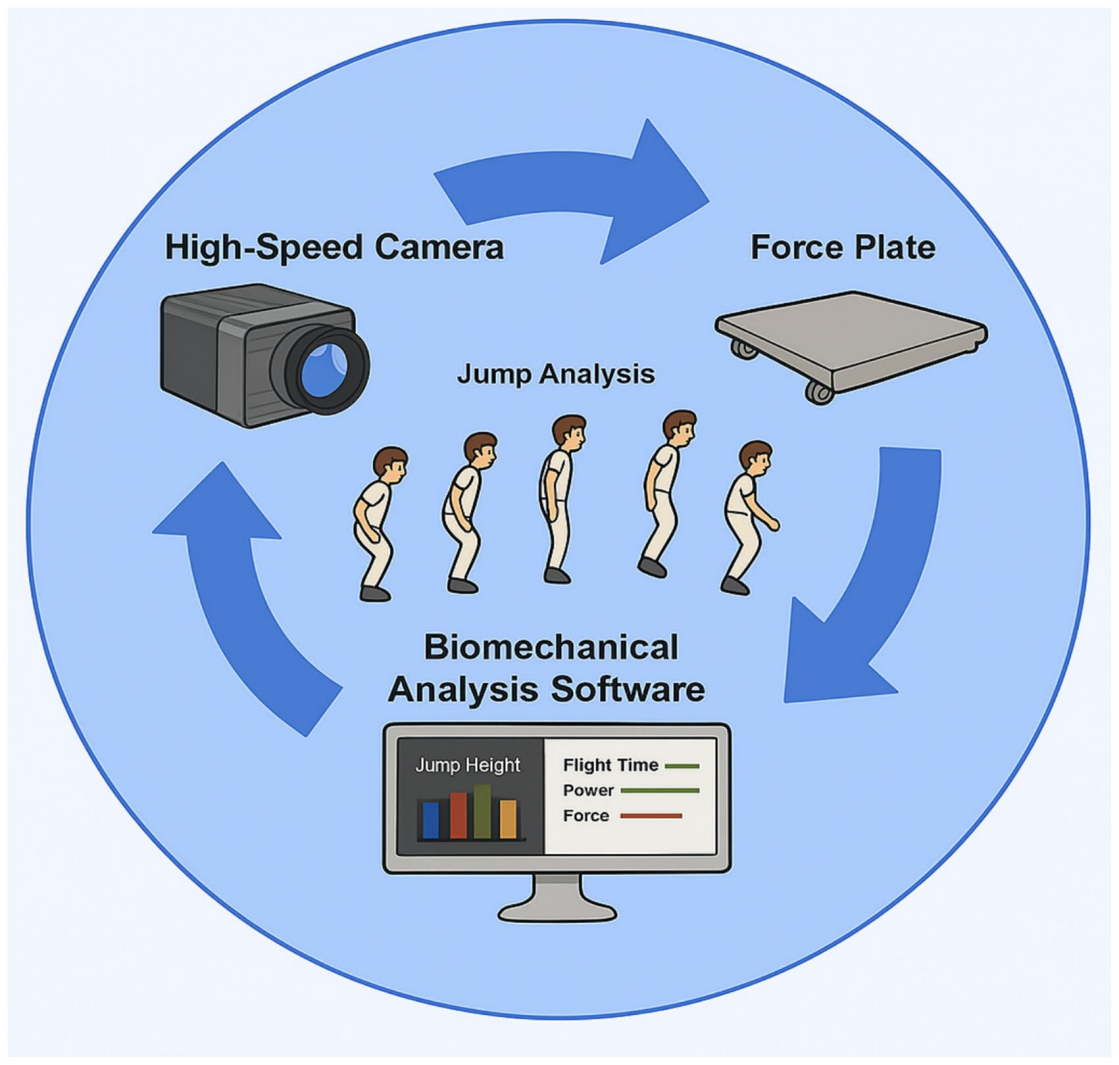
Technical infrastructure of jump assessment at the ZKN Dresden.

The jump assessment in Dresden is carried out on a force plate manufactured by Advanced Mechanical Technology Inc., (AMTI, Watertown, MA, USA, AccuPower-O.), which measures three-dimensional ground reaction forces (Fx, Fy and Fz) and force moments (Mx, My and Mz). A high-speed camera (Basler acA1300-200uc) is additionally used for qualitative video analysis. Using a special biomechanical analysis software (AccuPower Solutions, version 1.5.4.2082. Watertown, MA, USA), ground reaction curves from the force plate can be visualised and over 90 biomechanical parameters can be identified and evaluated. The multi-dimensional jump assessment shown in Figure 2 with evaluation of jump parameters, ground reaction curves and video recordings provide an assessment of motor function and allows the identification of MS motor symptoms like strength and balance deficits, ataxia, spasticity and coordination deficits (Figure 2).

### 4.1 Jump metrics

The collected jump parameters include kinetic (e.g., maximum force and power), performance (e.g., jump height) and temporal (e.g., flight time) parameters. An important consideration in the extraction of suitable jump parameter for MS was to analyse not only force parameters, but also time-based parameters, as these are more indicative of neuromuscular performance (65, 66).

Jump parameters such as jump height (ICC = 0.932) and maximal concentric force (ICC > 0.935) demonstrate good to excellent reliability (67, 68). Similarly, the jump parameter power shows acceptable reliability in a recent study by Anicic et al. (69) (ICC > 0.970). Furthermore, it has been generally argued that parameters from the eccentric phase exhibit lower reliability compared to those derived from the concentric phase (68, 69). The duration of the flight phase (flight time) is generally accepted as the most reliable parameter (ICC = 0.934) when considering the temporal parameters (68). On the other hand, the duration of the landing phase and the overall jump duration have not been extensively studied. Jump parameters such as flight-to-contraction time-ratio and reactive strength index (RSI) showed similar between-day reliability (all good-excellent based on CV% results) and share an almost perfect positive relationship (70). These two parameters provided valuable insight into the neuromuscular system for explosive and reactive performance characteristics of the lower extremities and can be used to monitor neuromuscular fatigue (70, 71).

### 4.2 Jump metrics and biomechanic factors in pwMS

These findings on the validity and reliability of the described jump parameters mostly are based on healthy people and athletes. Due to the fact that jump assessment is a new evaluation approach in the field of MS, there are no evidence on the validity and reliability of the jump parameters in MS; previous studies in pwMS have used the most common and reliable jumping parameters used in sports science (14, 24, 72). One of our previous study focused on analyzing SLCMJ in pwMS, we found that especially the two jump parameters jump height and negative power are suitable to detect differences between pwMS and healthy controls (HC) as well as differences between the dominant and non-dominant leg (24). These results are consistent with our two previous studies that investigated the CMJ in MS and found that performance parameters (i.e., jump height, RSI) and kinetic parameters (i.e., negative power, peak force, force at zero velocity) were particularly suitable for identifying group differences between pwMS and HC (14, 22). The correlation between EDSS and some jump parameters shows initial indications that the jump parameters are suitable for detecting neuromuscular deficits in MS (14, 24).

In previous studies we determine that CMJ performances in pwMS are characterized by a significantly lower force at zero velocity in the eccentric phase, in which participants squat to the lowest COM. Overall, the braking phase was significantly longer in pwMS. To achieve a rapid change between eccentric and concentric phases, a high eccentric force is necessary to develop an even higher concentric force. The low eccentric force in pwMS is therefore followed by a significantly lower peak force. This suggests a reduction in the strength of the lower limbs in pwMS, even in those with normal muscle strength according to the British Medical Research Council Rating Scale. In the eccentric to concentric phase ratio, pwMS demonstrated an inefficient SSC, as indicated by an increased brake to propulsive impulse ratio (14).

Nevertheless, future studies are needed to investigate the relation between the jump parameters and other functional assessments such as balance and gait assessments. With these aims - in future studies - the Dresden Protocol of Multidimensional Walking Assessment (DMWA) Dresden could provide a range of functional assessments at different levels (64). In addition to established measurements of walking speed (T25FWT) and endurance (2-min walk test), the DMWA also includes balance measurements (Romberg open and closed eyes) and a detailed assessment of gait quality.

## 5 An example of clinical implementation: the Dresden protocol for jump assessment in MS

In clinical practice, a combination of complementary jump tests may be useful, as no single assessment can capture the complexity of lower limb neuromuscular function. Since 2021, in the context of establishing the mobility laboratory in Dresden, we have explored which jump assessment methods appear feasible both for a specialized MS center and for routine neurological practice. To this end, we convened an expert group of neurologists, physiotherapists, biomechanical engineers, and MS nurses to define a feasible yet detailed jump assessment protocol for a center caring for more than 1,500 patients per year. With the aim of comprehensively capturing the relevant dimensions of neuromuscular function in pwMS, we combined functional assessments (i.e., jump assessment) with standard clinical outcome measures (e.g., EDSS) and patient reported outcomes (PROs). In addition to the EDSS and jump assessment, patients’ self-assessment via PROs is essential for evaluating treatment effects and disability progression in MS. We therefore used the Godin Leisure-Time Exercise Questionnaire (GLTEQ) as a validated PRO to quantify physical activity in pwMS (73). As several sport-specific studies have shown that high levels of physical activity have a positive effect on jumping performance in adults, it is important to consider physical activity as a confounding factor (74, 75). Seamless integration into the clinical workflow was a key criterion in developing the jump assessment protocol.

**Figure 3:**
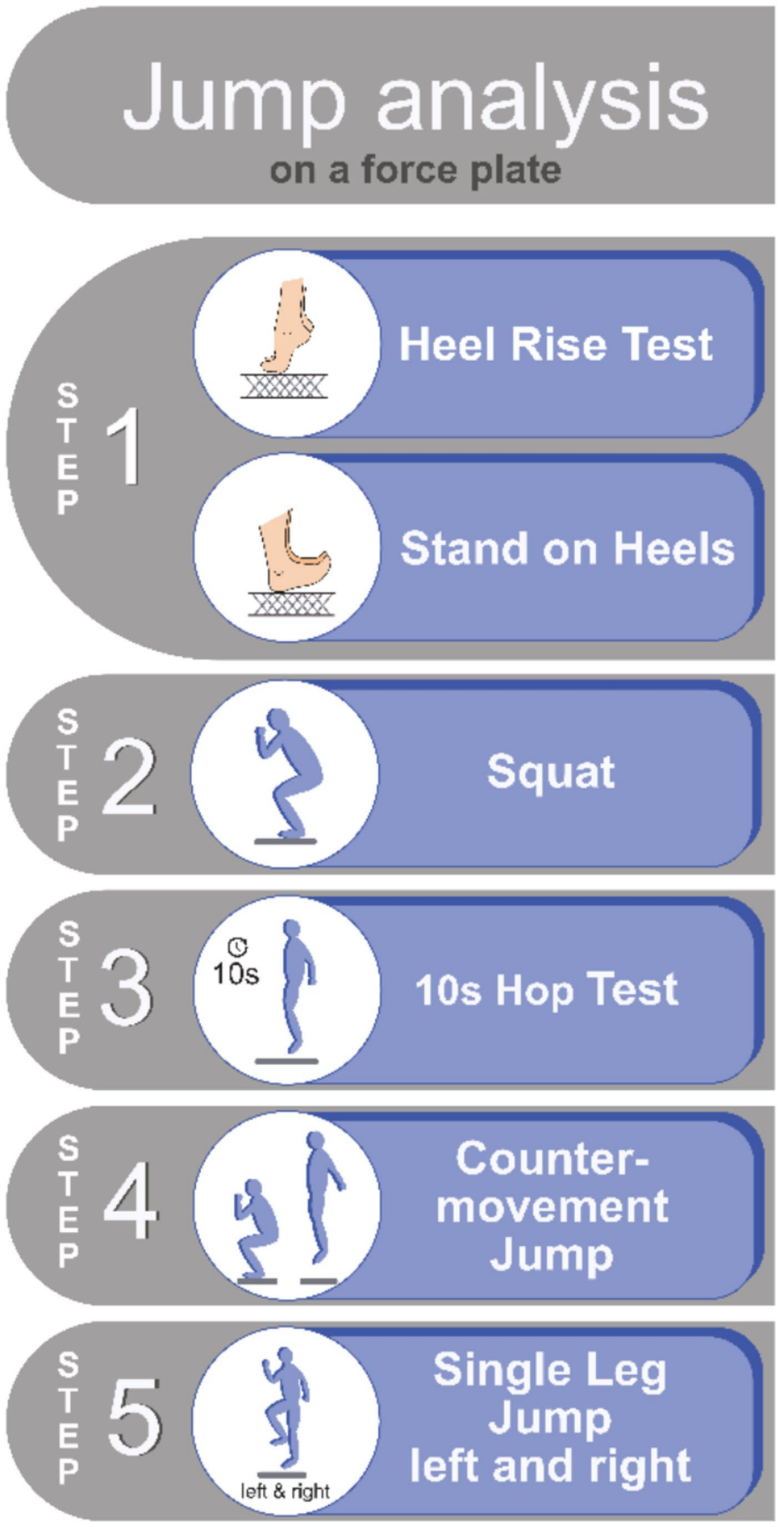
Dresden protocol for jump assessment in MS at ZKN Dresden.

The jump assessment is performed on a force plate using a stepwise, adaptive model tailored to the patient’s level of disability, with gradually increasing effort and task difficulty (Figure 3). This approach allows a broad spectrum of pwMS, including those with more advanced disability (up to an EDSS of 6.0), to participate in the testing. Pre-tests such as heel rise, heel stand, and squats provide initial information on general movement patterns, balance, proprioception, and muscle strength required for jumping. These are followed by three standardized jump tests with increasing difficulty: the 10-s hop test (10SHT), the countermovement jump (CMJ), and the single-leg countermovement jump (SLCMJ) (Figure 3).

### 5.1 Heel rise, heel stand and squat test in MS

The heel rise test, heel stand, and squat test are performed as pre-tests at the beginning of the standardized jump assessment in Dresden. In the heel rise test, patients are instructed to lift their heels off the ground bilaterally with maximal effort for three repetitions while keeping their hands on their hips. The heel stand test is analogous: patients raise their forefeet on both legs with maximal effort for three repetitions, minimizing compensatory trunk movements. In the squat test, patients perform three repetitions of a squat to approximately 90° knee flexion and return to the upright position without using arm swings. These tests help identify neuromuscular weaknesses that may impair jump performance and provide information on stability and balance, which are critical for fall prevention during dynamic tasks. All three pre-tests are multi-joint movements that require coordinated strength and mobility throughout the kinetic chain (76). Moreover, they are functionally relevant, reflecting everyday activities such as stair climbing and sit-to-stand transfers, and thus offer a comprehensive overview of a patient’s neuromuscular strength, balance, and overall capacity to perform jumping activities.

### 5.2 10-s hop test (10SHT) in MS

The 10SHT is a bipedal plyometric test to assess rapid stretch–shortening cycle performance, explosive lower-limb power and neuromuscular control during repeated hops in 10 s. It challenges strength, coordination and temporal precision under high-frequency loading. The hop tests have been highly recommended for evaluating sport-specific performance in healthy athletes and age-related muscle activation profiles (77, 78). To date, no studies have investigated the 10SHT in pwMS. Further studies could investigate whether the 10SHT is suitable for detecting lower limb neuromuscular deficits and motor fatigability in MS. A common symptom in pwMS is motor fatigue, a subjective lack of physical energy that is perceived as interfering with usual and desired activities (79). The objective measurement of the rate of change in motor performance after physical activity is defined as motor fatigability (80). According to a review by Severijns et al. (81) there is currently no gold standard for assessing motor fatigability in pwMS. Most studies (29/48) used maximal isometric single-joint contractions, although studies analysing motor fatigability during functional activities in more complex tasks, may be more relevant in pwMS (81, 82). One of the functional measurements of motor fatigability that has been widely studied is the 6-min-walk-test (6MWT), where pwMS showed increased objective walking fatigability compared to healthy subjects (83, 84). Although the 6MWT has been shown to be suitable for detecting motor fatgability in MS, the time required for the 6MWT and the burden on pwMS, particularly in those with increased disability, are important concerns in the clinical setting (85, 86). To be able to use a protocol to detect lower limb motor fatigability in a clinical setting, the protocol should be feasible, quick, easy to interpret, and reliable (81). Broscheid et al. found in their study that more intensity assessments are necessary for pwMS with low disability to detect motor fatigability, because the degree of disability is an important factor for the extent of motor fatigability (87, 88). With this aim of using such a protocol in clinical setting for evaluating motor fatigability in MS with low disability, we are currently investigating the 10SHT. For the 10SHT in Dresden, patients were instructed to jump bipedal as high and as fast as possible in 10 s without swinging their arms. An important additional instruction was to touch the force plate with the heels after each jump.

### 5.3 Countermovement jump (CMJ) in MS

The CMJ is a bipedal vertical jump test often used in sports performance diagnostics to assess motor function of the lower extremities, particularly explosive strength and neuromuscular function (89). The CMJ is a highly demanding task for pwMS as it strongly challenges the neuromuscular system by combining strength, balance, coordination and muscle timing in one assessment (12).

During the CMJ, patients are instructed to jump as high as possible in a bipedal position with no arm swing using maximum force. Excluding the arm swing removes the additional force generated by the arm movement and ensures that the jump is primarily powered by the strength of the lower limbs. This allows for a more accurate assessment of explosive power, neuromuscular function and coordination of the lower limb. In Dresden the pwMS performed the CMJ three times with a rest of 5 s and the average of the three jumps were used for analysis. This approach supports the findings of the meta-analysis by Claudino et al. (90) who found that average CMJ values were more sensitive than the highest CMJ values when monitoring the effects of fatigue and supercompensation on performance. In all comparisons between the highest and average CMJ performance, the average values were found to be more sensitive than the highest values when monitoring neuromuscular status (90).

Three studies examined the CMJ in pwMS (see Table 1). In MS the CMJ performance was characterized by significantly lower peak force in the concentric phase, lower force at zero velocity in the eccentric phase, longer braking phase, lower jump height and an overall inefficient SSC as indicated by increased RSI and FTCTR (14). With increasing disability (EDSS) in pwMS, it was also observed that CMJ performance decreased significantly. CMJ performance is known to be influenced by various anthropometric, physiological and biomechanical factors, mostly investigated in children and adult athletes (74, 75, 91, 92).

However, a recent study of 164 pwMS and 98 HC showed that age, gender and BMI had a significant effect on all CMJ performance parameters, flight time and negative and positive power in MS (24). With increasing age and BMI, CMJ performance decreased and was higher in men than in women.

The CMJ on a force plate is a new objective assessment tool in MS that appears to be able to detect subthreshold neuromuscular deficits in pwMS who have normal motor, cerebellar and sensory Functional System Score according to the EDSS (14). Using the CMJ as part of the motor diagnosis in MS can identify deficits in eccentric and concentric muscle activity. Longitudinal studies on CMJ performance in pwMS are needed to investigate whether the CMJ can be used as a standardized measure for the mid- and long-term assessment of disease progression and treatment response in MS.

### 5.4 Single-leg countermovement jump (SLCMJ) in MS

The SLCMJ is a unilateral vertical jump test often used in sports performance diagnostics to characterize lower-limb functional ability and quantify limb asymmetries by measuring unilateral strength (40, 93). A large number of studies confirm the high validity and reliability of the SLCMJ on a force plate to objectively assess the symmetry of neuromuscular function (44, 94). Variations in force–time-related jump performances between dominant and non-dominant legs in SLCMJ are indicative of functional strength asymmetries (95). Strength asymmetry is defined as a relative difference of > 10–15% in maximum muscle power between the contralateral muscle groups (96). A large number of studies have investigated strength asymmetries in various muscle groups of the lower extremity in pwMS (97–99). One review reported strength asymmetries in plantar flexion, dorsiflexion, knee extension and knee flexion in pwMS (100).

Study results also suggest that strength asymmetries in pwMS lead to increased muscle energy costs, fatigue and impaired gait and balance compared to healthy controls (99). In their study, Chung et al. also highlight a correlation between strength asymmetry, postural stability, walking speed and fatigue (97). Comparison of unilateral neuromuscular performance using the SLCMJ in pwMS can provide findings on strength asymmetries, intramuscular coordination deficits and dynamic balance disorders and offers an approach for appropriate neurorehabilitative training interventions. During the SLCMJ in Dresden, pwMS are instructed to jump as high as possible on one leg without arm swing for three times with maximum force and a rest of 5 s. In a first study of 126 pwMS and 96 HC, the SLCMJ was investigated to detect subclinical lower limb asymmetries in pwMS (24) (see Table 1). The results of the study suggest that pwMS with normal muscle strength according to manual muscle function tests have significantly reduced SLCMJ performance compared to HC. In both pwMS and HC, jumping performance differed significantly between the dominant and non-dominant leg, but with a significantly higher effect size in pwMS, indicating that pwMS had a greater difference between the sides of the leg than HC. These results suggest that pwMS not only have physiological differences, but also subclinical asymmetries in neuromuscular function at an early stage of disability, as detected by the SLCMJ. These results of reduced single leg jump performance in MS are consistent with other neurological diseases like Wilsons’s Disease, Spastic Cerebral Palsy and Stroke patients (101–103). Patients with Wilsons disease showed a second, sharp, initial “impact” force peak during ground contact in addition to the usual “active” force peak in single leg jumps. The presence of these sharp initial “impact” peak in the ground reaction force curves of single leg jumping may indicate a mild deficit of limb/trunk coordination and subclinical cerebellar impairment (102). The majority (82%) of patients with Wilson’s disease who participated in this study, as well as patients with spastic cerebral palsy, stroke patients with lower limb hemiplegia and pwMS from other studies, were able to perform single-leg jumps (24, 101–103). This indicates that patients with neurological disorders are able to perform complex motor tasks, such as single-leg jumps.

In order to enhance the understanding of SLCMJ in MS, it is essential to consider the inclusion of longitudinal studies that monitor the changes in SLCMJ performance throughout the disease progression. Such studies could assist in determining whether SLCMJ can serve as an early indicator of MS progression and for lower limb asymmetries. In addition, the potential of the SLCMJ as an assessment tool to evaluate the effectiveness of rehabilitation interventions or targeted training programmes aimed at reducing strength asymmetries should be investigated, as it could provide a practical approach to improving overall functional performance in pwMS (101).

## 6 Discussion

In this narrative review, we sought to summarise current approaches to jump assessment in MS, evaluate their suitability for specialised clinical practice, and present the jump assessment protocol implemented in Dresden. Despite substantial progress in the assessment of neuromuscular function in pwMS, subtle impairments remain difficult to detect with traditional clinical tools, as their complexity and early onset often elude routine neurological examination. Sensor-based approaches such as vertical jump assessment appear particularly promising for identifying subclinical deficits in the early stages of MS, when therapeutic interventions are most effective. The multidimensional nature of jump analysis with kinetic, temporal, and performance parameters, ground-reaction force curves, and video analysis offers clinicians deeper insight into strength, coordination, balance, and motor control in pwMS. These insights may be critical for detecting neuromuscular dysfunction that would otherwise remain clinically invisible.

However, several challenges still hinder the broader implementation of jump assessment in clinical practice. A key barrier is the technical complexity of the method, which traditionally relies on specialised, research-grade multiaxial force plates regarded as the gold standard for accurately measuring ground reaction forces during jumping. Emerging technologies, including smartphone-based applications, micro-laser systems, and accelerometer-based devices, aim to deliver comparable accuracy in a more accessible and cost-effective manner, potentially facilitating wider use of jump assessment beyond specialised centres (104, 105). In addition, interpretation of the comprehensive measurement jump parameter is challenging and requires standardised protocols and specially trained professionals. Integrating jump assessment into existing clinical processes and documentation systems could also increase the administrative burden. Previous studies on jump assessment in MS indicate that participating pwMS usually have low disability levels (EDSS < 3) (14, 22, 23). This suggests that jump assessment is particularly suitable for the early stages of the disease, before motor systems are markedly affected. At the same time, it underscores an important limitation: in patients with manifest gait impairment or more pronounced motor deficits, established tools such as multidimensional gait analysis are more appropriate. A recent study by Trentzsch et al. (106) shows that instrumented gait analysis should be integrated into routine clinical care to improve the precision of motor phenotyping and to guide phenotype-specific rehabilitation. The authors further emphasize the need for longitudinal protocols to monitor gait changes over time, identify early indicators of disease progression, and evaluate targeted interventions. Combining sensor-based gait measures with cognitive assessments, disease-related variables, and patient-reported outcomes may further enhance the clinical utility of gait phenotyping and support a more individualized approach to MS management. In line with these advances in gait phenotyping, the goal should be to establish and implement jump assessment as an equally robust, clinically integrated tool for the early and sensitive detection of neuromuscular dysfunction in pwMS. Another challenge is patient acceptance, as jumping tasks can be demanding, particularly for individuals with physical limitations. Moreover, the development and implementation of a jump assessment protocol are complex and must always be interpreted alongside other clinical and paraclinical parameters. Nevertheless, in addition to promising study results, jump analysis in MS has shown very good clinical feasibility (sample sizes: pwMS N ≥ 125, healthy controls N ≥ 75) with a low risk of injury. This was corroborated by a study of 157 pwMS in which patient experiences with jump analysis (consisting of 10 SHT, CMJ, SLCMJ) were evaluated using a paper-based questionnaire (Patient-Reported Experience Measures—PREMs) (see Table 1) (23). The SLCMJ was experienced as the significantly most difficult and exhausting jump test, followed by the 10SHT and the CMJ. PwMS who experienced the CMJ as easy, not exhausting, and safe were associated with higher CMJ performance, especially in peak power, flight time, and jump height. Overall, pwMS showed an positive experience with a high level of comfort, perceived safety, usefulness, and integration of the assessment into clinical care. Most participants (60–70%) found jump analysis easy to perform and not very strenuous, and the majority (79.2%) stated that they would like to undergo jump assessment once per year. These findings are crucial for the successful implementation of sensor-based jump assessment—particularly the CMJ—in clinical practice as a tool for continuous, patient-centered monitoring in early MS.

For successful integration of jump assessment into standardized, patient-centered MS care, it is essential to systematically collect patient-reported experiences while considering relevant influencing factors (e.g., clinical parameters, demographic and anthropometric characteristics, physical activity). This approach supports long-term acceptance, sustainable adherence, optimisation of care pathways, and quality assurance. Jump assessment can be conducted as a comprehensive protocol or via selected individual tests: the CMJ primarily evaluates explosive strength and neuromuscular function; the SLCMJ assesses inter-limb asymmetries; and the 10SHT examines strength, coordination, and temporal precision under high-frequency loading. With the ZKN Dresden force-plate–based jump assessment, it is possible to obtain detailed, objective, and metric information on lower-limb neuromuscular function, providing a potentially sensitive approach for detecting functional impairments. In addition, jump assessment may help to strengthen the role of patient-centered, evidence-based outcomes in routine clinical practice. By revealing subtle motor deficits at an early stage, it may support timely, event-driven therapeutic interventions, potentially preventing further progression of impairment and improving the efficiency and effectiveness of patient monitoring and care (8).

Another important limitation is that the Dresden protocol for jump assessment in MS presented in this narrative review is based on the implementation approach of a single centre, derived from available literature and local clinical experience. It should therefore not be interpreted as a validated, evidence-based standard, but rather as a structured proposal for practice that requires further prospective, MS-specific validation.

On the other hand, it is intended to integrate jump assessment into the documentation process of long-term MS therapy in order to better record the course of the disease and to promote the shared decision-making process of therapy optimisation between physician and patient. These innovative methods of analysis allow detailed phenotyping of the MS cohort and thus more efficient treatment methods (16). Appropriate interventions are required for the treatment of neuromuscular deficits diagnosed using jump assessment. Current reviews and guideline recommendations showed, that pwMS who participate in neurorehabilitation programs improve their quality of life and are more independent in their activities of daily living (107, 108). In addition, several symptoms associated with MS are positively influenced by exercise, e.g., neuromuscular function (109), walking ability (110), depression (111) and cognitive performance (112). Appropriate neurorehabilitation programs for MS include resistance training that leads to improvements in muscle strength, neural drive and spinal motor neuron efferent motor performance in pwMS (113, 114). However, it is known that maximal strength training is more demanding on the neuromuscular system, muscle fiber recruitment and neural drive than other forms of strength training (115). As a result of these characteristics and the reduction in peripheral pro-inflammatory cytokine levels, high intensity strength training appears to be more appropriate than resistance training (116). Based on these findings, high training loads such plyometric strength training may be a key component of muscle strength training to positively influence a variety of MS symptoms. Previous studies that investigated plyometric strength training in neurological patients (Stroke, traumatic brain injury, Parkinson’s disease, MS), showed a high level of safety and feasibility in 88% of cases, as well as a positive effect on muscle strength and performance (117). To date, there are no other studies that have investigated plyometric strength training in MS in comparison with other training programs. Future studies should investigate the effects of plyometric strength training that includes jumping exercises such as the jumps from the jump assessment.

### 6.1 Implication of research

Current evidence highlights several implications for future research on jump assessment in pwMS. Based on the currently available evidence, jump assessment may be particularly relevant in pwMS with low disability, especially the CMJ as a subthreshold test complementary to the neurological EDSS in the early stage of MS. Performance parameters (e.g., jump height and RSI) and kinetic parameters (e.g., negative power, peak force and force at zero velocity) were the most suitable jump metrics for detecting neuromuscular deficits in pwMS.

Due to the prevalence of cross-sectional study designs, longitudinal studies are required to establish whether jump parameters can be used to monitor disease progression, fall risk and patient-reported functional ability over time. Additionally, the role of jump assessment as outcome in rehabilitation requires further investigation. Therefore, future studies should investigate the responsiveness of the jump assessment to targeted interventions, such as resistance, balance and plyometric training, and compare its performance with established clinical and instrumented outcome measures. Finally, further research is required to establish its psychometric properties in pwMS particularly with regard to test–retest reliability, measurement error, and construct validity in relation to gait, balance, mobility, and muscle function.

## 7 Conclusion

In summary, jump assessment may represent a promising approach to multidimensional, precision rehabilitation in MS, potentially enabling more accurate symptom profiling and truly patient-centred monitoring. It could also support individualised optimisation of neurorehabilitative and therapeutic strategies, while also providing functional inputs for the development and refinement of our digital MS twin (16). While evidence from sports science and other populations supports the biological rationale of this approach, MS-specific validation is still required. Further research is needed to determine the psychometric properties, clinical usefulness and suitable applications of jump assessment in pwMS.
